# Retraction Note: SARI inhibits angiogenesis and tumour growth of human colon cancer through directly targeting ceruloplasmin

**DOI:** 10.1038/s41467-023-40067-6

**Published:** 2023-07-20

**Authors:** Lei Dai, Xueliang Cui, Xin Zhang, Lin Cheng, Yi Liu, Yang Yang, Ping Fan, Qingnan Wang, Yi Lin, Junfeng Zhang, Chunlei Li, Ying Mao, Qin Wang, Xiaolan Su, Shuang Zhang, Yong Peng, Hanshuo Yang, Xun Hu, Jinliang Yang, Meijuan Huang, Rong Xiang, Dechao Yu, Zongguang Zhou, Yuquan Wei, Hongxin Deng

**Affiliations:** 1 State Key Laboratory of Biotherapy/Collaborative Innovation Center for Biotherapy, West China Hospital, Sichuan University, Chengdu, Sichuan 610041 China; 2 Huaxi Biobank, West China Hospital, Sichuan University, Chengdu, Sichuan 610093 China; 3 Department of Thoracic Oncology, Tumour Center, West China Hospital, Sichuan University, Chengdu, Sichuan 610041 China; 4 Department of Immunology, Nankai University School of Medicine, Tianjin, 300071 China; 5 Department of Gastrointestinal Surgery, West China Hospital, Sichuan University, Chengdu, Sichuan 610041 China

Retraction to: *Nature Communications* 10.1038/ncomms11996, published online 29 June 2016

The Editors have retracted this article. Concerns were raised regarding Figures 3, 4, 5 and Supplementary Figures 1 and 6. Concerns include but are not limited to overlapping or duplicated figures as well as splices in western blots. The Editors therefore no longer have confidence in the results and conclusions of this article.

Author Rong Xiang agrees with this retraction. Author Hongxin Deng disagrees with this retraction. The other authors have not responded to correspondence regarding this retraction.

